# Connected Mental Health: Systematic Mapping Study

**DOI:** 10.2196/19950

**Published:** 2020-08-28

**Authors:** Nidal Drissi, Sofia Ouhbi, Mohammed Abdou Janati Idrissi, Luis Fernandez-Luque, Mounir Ghogho

**Affiliations:** 1 Department of Computer Science and Software Engineering United Arab Emirates University Al Ain United Arab Emirates; 2 National School For Computer Science Mohammed V University in Rabat Rabat Morocco; 3 Salumedia Labs Seville Spain; 4 Adhera Health Inc Palo Alto, CA United States; 5 TICLab International University of Rabat Rabat Morocco

**Keywords:** mental health, connected health, eHealth, mobile health, telehealth, mHealth, mobile phone, health informatics, review, interdisciplinary research, information technology, information systems

## Abstract

**Background:**

Although mental health issues constitute an increasing global burden affecting a large number of people, the mental health care industry is still facing several care delivery barriers such as stigma, education, and cost. Connected mental health (CMH), which refers to the use of information and communication technologies in mental health care, can assist in overcoming these barriers.

**Objective:**

The aim of this systematic mapping study is to provide an overview and a structured understanding of CMH literature available in the Scopus database.

**Methods:**

A total of 289 selected publications were analyzed based on 8 classification criteria: publication year, publication source, research type, contribution type, empirical type, mental health issues, targeted cohort groups, and countries where the empirically evaluated studies were conducted.

**Results:**

The results showed that there was an increasing interest in CMH publications; journals were the main publication channels of the selected papers; exploratory research was the dominant research type; advantages and challenges of the use of technology for mental health care were the most investigated subjects; most of the selected studies had not been evaluated empirically; depression and anxiety were the most addressed mental disorders; young people were the most targeted cohort groups in the selected publications; and Australia, followed by the United States, was the country where most empirically evaluated studies were conducted.

**Conclusions:**

CMH is a promising research field to present novel approaches to assist in the management, treatment, and diagnosis of mental health issues that can help overcome existing mental health care delivery barriers. Future research should be shifted toward providing evidence-based studies to examine the effectiveness of CMH solutions and identify related issues.

## Introduction

### Background

Mental health issues can decrease the quality of life [1,2], increase the risk of communicable and noncommunicable diseases, and contribute to both unintentional and intentional injury [3]. They may also cause, among other issues, lower educational achievements, substance abuse, and violence [4]. Mental illness is considered as “one of the main causes of unhappiness in the world. It produces nearly as much of the misery that exists as poverty does, and more than is caused by physical illness” [5]. On average, they reduce national income by 5% through unemployment, absenteeism, lowered productivity, and extra physical health care costs [6].

Although mental health issues constitute a global concern and threat, the mental health care industry is still struggling to overcome various barriers and reach people in need [7]. In low- and middle-income countries, more than 75% of people identified with serious anxiety, problematic mood changes, impulse control, or substance abuse disorders did not receive any care [8]. Among the barriers and challenges that threaten the mental health care industry are cost issues [9], shortage of mental health care providers, health plan barriers, lack of coverage or inadequate coverage [10], stigma, and poor mental health literacy [11]. Cultural orientations can, in some cases, be considered as a barrier for seeking mental help and access [12]. Mental health care delivery can also face barriers in complicated situations such as the recent coronavirus disease (COVID-19) global outbreak [13,14]. Such pandemics create global feelings of fear, worry, sadness, and anger and cause a global increase in stress and anxiety, especially for people with existing mental health problems [15,16], putting more load on health care institutions. They also create new obstacles to mental health care delivery as many people are in quarantine and several countries are in complete lockdown, making access to mental health care even more challenging [17].

Connected mental health (CMH), which refers to the use of information and communication technologies (ICT) to support and improve mental health conditions and mental health care, can help alleviate some of the aforementioned barriers [18]. This presents unlimited possibilities and opportunities to help overcome the challenges, barriers, and limitations of the mental health care industry [18].

CMH solutions are connected health solutions for mental health care. The term *connected health* is used to “encompass terms such as wireless, digital, electronic, mobile, and tele-health” [19]. Connected health has become an established research field in the past 5 years [20]. In this paper, we use the term CMH to refer to topics related to electronic mental (e-mental) health, mobile mental (m-mental) health, telemental health, and digital mental health, among others. CMH solutions have the potential to allow anonymous access to overcome care preventing stigma, access to care with minimal to no cost, reach remote areas, enhance patients’ engagement, and access information [21,22]. They also have the potential to deliver cost-effective mental health services [23]; overcome geographical barriers [24]; and provide a better understanding of mental illnesses’ development, recovery, symptom assessment, and monitoring through the use of technology [25]. CMH can use technology-based interventions such as video conferencing; telemental health [26]; mobile-based interventions [27,28]; internet-based solutions [29,30], which are necessary in situations such as global health pandemics; and novel digital data collection interventions [31,32]. In addition, CMH can improve several important areas of mental health care, including primarily information provision, screening, assessment, monitoring, intervention, and social support [22].

### Objectives

This paper presents the results of a systematic mapping study conducted to analyze the current research landscape of CMH literature in the Scopus database. To conduct the analysis, 8 mapping questions (MQs) were addressed, which allowed us to classify 289 selected publications indexed in Scopus according to their publication year, publication source, research type, contribution, empirical type, mental health issues, and targeted cohort.

## Methods

### Overview

This study follows the mapping process proposed by Petersen et al [33]. This process covers the selection of relevant publications, the construction of a classification scheme, and a systematic mapping of publications. The principal objective of a systematic mapping study is to structure a research area and provide an overview of the available literature, primarily by investigating the covered topics and classifying the available contributions [34].

### MQs

Textbox 1 presents the 8 MQs and their rationale. The MQs were defined to provide an overview and a structured understanding of the existing CMH literature in the Scopus database.

**Mapping questions.**

MQ1 (mapping question)

- How has the frequency of publications addressing CMH (connected mental health) changed over time?

MQ2

- Which publication channels are the main target for CMH research?

MQ3

- What are the research types of studies addressing CMH?

MQ4

- What are the contributions of published CMH studies?

MQ5

- Are CMH studies empirically validated or evaluated?

MQ6

- What are the mental health disorders addressed in CMH literature?

MQ7

- Who are the target audience in CMH studies?

MQ8

- In which countries were the selected empirically evaluated studies conducted?

### Search Strategy

The search was conducted in the Scopus database, which is considered to be the largest indexation database that includes articles from various disciplines such as engineering, medicine, business, and computer science [35].

The aim of the selection process was to identify the articles that are most relevant to the objective of this mapping study. To further focus the search and include relevant studies, the search was focused on the titles of the publications.

The following search strings were applied to the titles of the publications indexed in Scopus:

- “e-” AND “mental” AND “health”
- (“Mobile” OR “M-”) AND “mental” AND “health”
- “Digital” AND “mental” AND “health”
- “Connected” AND “mental” AND “health”
- “Tele” AND “mental” AND “health”

The search strings were formulated to include a broad selection of literature and were not combined in one search string for the purpose of identifying the number of results for each term separately. The search for publications was conducted on January 23, 2020.

### Papers Selection

The first author (ND) retrieved candidate papers from the results of the search and entered information in an Excel (Microsoft Corporation) file that was shared with the second author (SO) for revision. The two authors examined the title, abstract, and keywords and then commented on whether the paper was to be included in or excluded from the selection according to the inclusion and exclusion criteria (EC).

The inclusion criterion was limited to publications that addressed an aspect of CMH, and the studies that satisfied any one of the following EC were eliminated:

- EC1: studies published after 2019 to construct a clear trend of publication
- EC2: studies not published in English, as there is a clear dominance of the English language in the international communications, science, and literature [36,37]
- EC3: papers published as notes, editorials, or letters
- EC4: publications not addressing mental health issues
- EC5: publications not addressing the use of technology for mental health.

**Figure 1:**
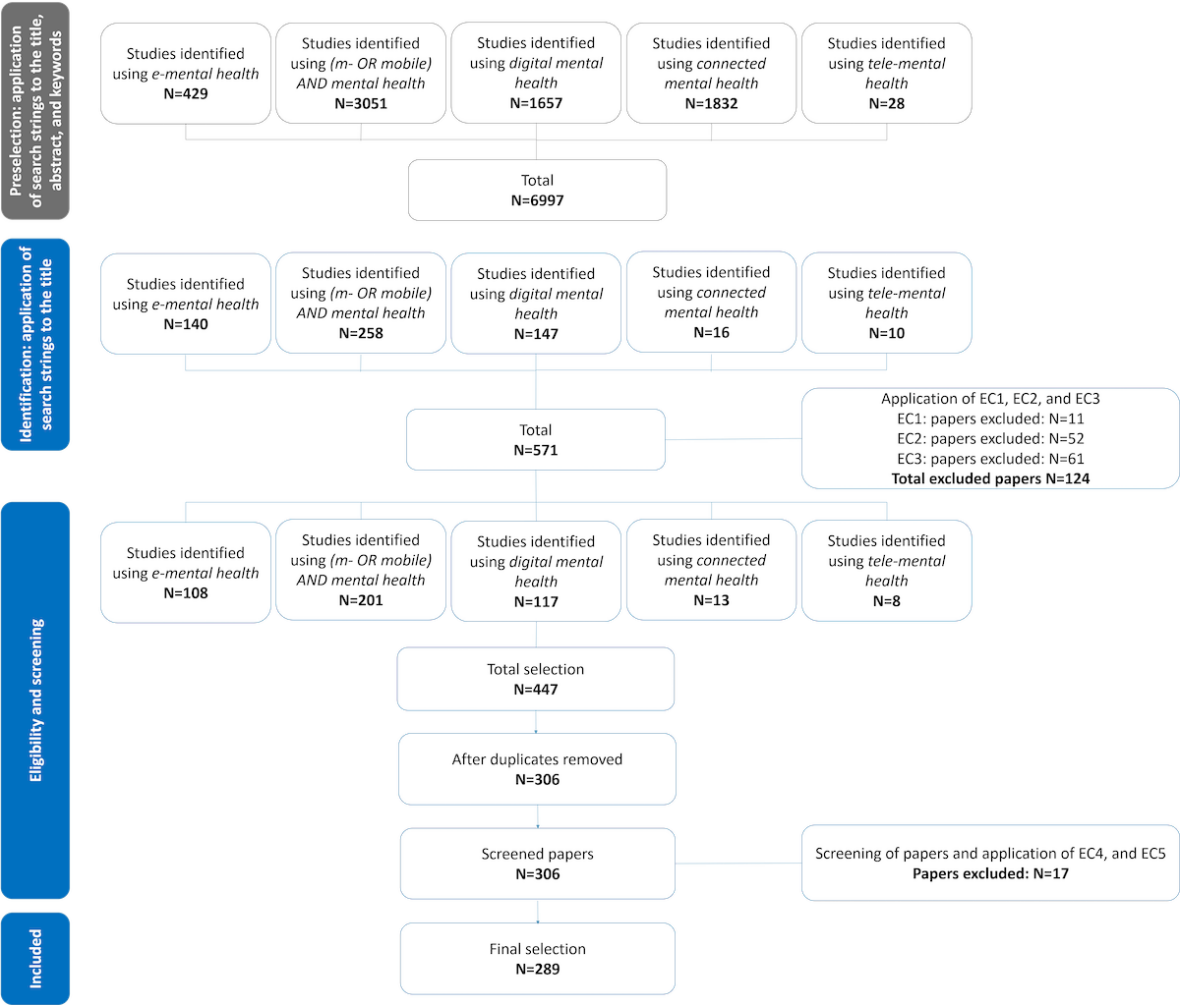
Selection process. EC: exclusion criteria; e-mental: electronic mental; tele-mental health: telehealth for mental health.

Figure 1 shows the selection results. A total of 289 papers (out of 571 candidate studies) were included in the final selection. EC 1, 2, and 3 were applied in the Excel file. Checking for EC 4 and 5 and the screening of studies were conducted by inspecting the abstracts and, in some cases, the full texts of the candidate studies.

### Data Extraction Strategy

The extraction of data from the selected studies was focused primarily on providing answers to the MQs according to the criteria presented in Textbox 2.

**Classification criteria.**

MQ1 (mapping question)

- Identifying the publication year and term used can assist in suggesting the publication trend. The main term of each study was not based on the results of the selection process but was based on an analysis of the content of the publications

MQ2

- Identifying the publication channel and the publication source of each study

MQ3

- Research types can be classified as follows: (1) solution proposal: a solution to an existing problem, either novel solution or a significant extension of an existing solution; (2) review: analysis of the existing literature; (3) exploratory study: conducting research and investigating on a specific aspect; (4) opinion paper: these papers express the personal opinion of the author on a specific technique not relying on related work or research methodologies; (5) validation research: this type of paper investigates novel solutions that have not been implemented yet; (6) evaluation research: conducting a study on an implemented solution by identifying how the solution has been implemented in practice, the benefits and drawbacks of the implementation, and relevant problems in the field; (7) feasibility study: an analysis of relevant factors of success of an intervention to determine the likelihood of its implementation successfully; and (8) books and book chapters

MQ4

- The contributions of the selected studies can be classified as follows: (1) tool-based techniques: techniques based on software or devices used to accomplish a connected mental health (CMH)–related task; (2) model: a system that allows structured utilization of CMH solutions; (3) method: development or design of an approach by creating a series of steps that can assist in utilizing CMH solutions; (4) guidelines: a set of rules or recommendations to be followed during the course of an action related to CMH; (5) framework: a real or conceptual structure intended to support and guide the creation of CMH-related contributions; (6) protocol: a set of rules guiding use of technology for mental health care; (7) perspectives and attitudes toward CMH: results on perspectives, attitudes, acceptance, and preferences toward CMH in general or in a specific group of people; (8) advantages and challenges of CMH: views regarding the advantages, benefits, risks, challenges, and limitations of CMH; and (9) other: results of general research on the efficacy, effectiveness, and usability of CMH interventions

MQ5

- The empirical types can be classified as follows: (1) experiment: testing a hypothesis by creating a situation with controlled conditions; (2) case study: testing a solution in real life, with the purpose of gathering new information and detecting potential issues by analyzing real cases; (3) questionnaire: a set of specific, easy to answer questions targeting an audience to collect large amounts of data; (4) interview: a conversational method based on asking specific questions to gather in-depth, precise, and meaningful data; (5) mixed methods: using more than one empirical evaluation method; (6) focus group: investigating results and feedback in a relatively small group of participants; (7) other: all empirical evaluation methods that do not belong to any of the categories above; and (8) none: all solutions that were not validated empirically were classified as theories

MQ6

- Identifying the mental problem addressed in each study

MQ7

- Identifying the targeted cohort group

MQ8

- Identifying the country or region where the study has been conducted. Nonempirically evaluated studies are usually general, whereas empirically evaluated studies focus on specific groups and countries or regions. Therefore, for this question, we focus on the latest

### Synthesis Method

The synthesis method used in this study consisted of the following steps:

- Reading and analyzing the 289 selected studies to extract information presented in the *Data Extraction Strategy* subsection.
- Classifying the studies by enumerating the number of publications per MQ. It should be noted that selected publications addressing more than one mental health issue (MQ6), more than one cohort group (MQ7), and more than one country (MQ8) were counted in each category.
- Presenting the classification results in figures and charts, such as bubble plots, as visualization of the results facilitated their analysis.
- Presenting a narrative summary to describe the principal findings.

### Compliance With Ethical Standards

This paper does not contain any study with human participants or animals.

## Results

This section summarizes the results of the MQs. The results of the mapping study can be found in Multimedia Appendix 1.

### MQ1: How Has the Frequency of Publications Addressing CMH Changed Over Time?

**Figure 2:**
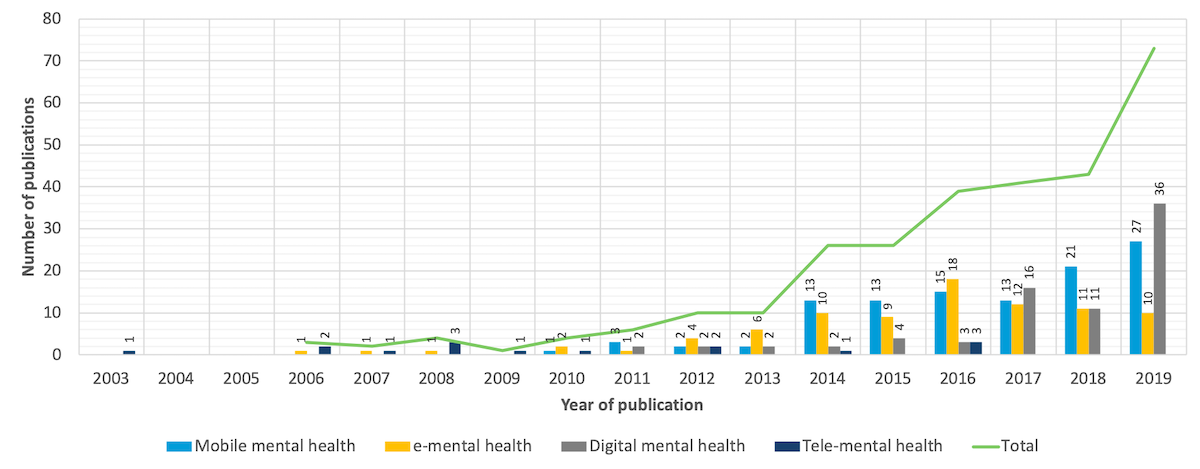
Connected mental health publication trend. e-mental: electronic mental; tele-mental health: telehealth for mental health.

Figure 2 shows the publication trend per term used in the selected papers: mobile or m-mental, e-mental, digital, and telemental health. This shows a significant increase in interest in the field of CMH research in the past decade. The publication trend began with fewer than 5 publications per year until 2011. We estimate that the trend of publication will continue to increase in 2020, particularly owing to the increasing interest in mental health during the recent COVID-19 pandemic.

### MQ2: Which Publication Channels Are the Main Target for CMH Research?

A total of 3 publication channels have been identified from the selected studies: journals, conferences, and books. Overall, 82.3% (238/289) of the selected papers were published in journals, 11.4% (33/289) were published in conference proceedings, and 6.2% (18/289) were published in books. A total of 39 of the selected studies were retrieved from the *Journal of Medical Internet Research*. Table 1 presents publication sources that published two or more selected studies.

It is worth noting that there might be relevant studies conducted in e-mental health and mobile health companies intended for internal use only and not published in scientific venues. The findings of such studies might offer new interesting insights and might help evolve the CMH field.

**Table 1:** Publication sources that published more than one selected publication.

Publication sources	Publications, n	References
Journal of Medical Internet Research	39	[38-76]
e-Mental Health	8	[77-84]
Journal of Mental Health	7	[18,25,85-89]
BMC Psychiatry	7	[90-96]
Psychiatric Services	7	[22,97-102]
Frontiers in Psychiatry	7	[103-109]
Studies in Health Technology and Informatics	6	[110-115]
Current Treatment Options in Psychiatry	6	[116-121]
Journal of Technology in Human Services	5	[122-126]
Professional Psychology: Research and Practice	4	[127-130]
Australian and New Zealand Journal of Psychiatry	4	[131-134]
Internet Interventions	3	[135-137]
Current Psychiatry Reports	3	[138-140]
Asian Journal of Psychiatry	3	[141-143]
Australian Family Physician	3	[144-146]
Evidence-Based Mental Health	3	[147-149]
Lecture Notes in Computer Science (including subseries Lecture Notes in Artificial Intelligence and Lecture Notes in Bioinformatics)	3	[150-152]
PLoS ONE	3	[153-155]
International Journal of Mental Health Nursing	3	[156-158]
Telemedicine and e-Health	3	[159-161]
Psychiatric Times	3	[162-164]
Frontiers in Public Health	3	[165-167]
Health Informatics Journal	3	[168-170]
Indian Journal of Psychological Medicine	3	[171-173]
JAMA Psychiatry	2	[174,175]
Journal of Psychosocial Nursing and Mental Health Services	2	[176,177]
Journal of Physics: Conference Series	2	[178,179]
BMC Medical Informatics and Decision Making	2	[180,181]
Psychiatry (New York)	2	[182,183]
Conference on Human Factors in Computing Systems—Proceedings	2	[184,185]
Journal of Affective Disorders	2	[186,187]
Cyberpsychology, Behavior, and Social Networking	2	[188,189]
Journal of Psychiatric Research	2	[190,191]
European Journal of Psychotraumatology	2	[192,193]
IEEE International Symposium on Computer-Based Medical Systems—Proceedings	2	[194,195]
Advances in Mental Health	2	[196,197]
The Digitization of Healthcare: New Challenges and Opportunities	2	[198,199]
Australian Psychologist	2	[200,201]

### MQ3: What Are the Research Types of Studies Addressing CMH?

**Figure 3:**
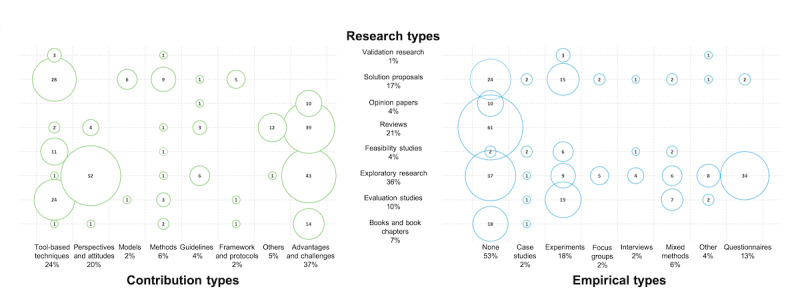
Bubble graph associating the research types with the empirical types and the contribution types.

Figure 3 presents the research types identified in the selected papers. The largest number of the selected publications were exploratory research (104/289, 35.9%), followed by reviews (61/289, 21.1%) and solution proposals (49/289, 16.9%); however, 10.0% (29/289) and only 1.3% (4/289) were evaluation studies and validation research, respectively.

### MQ4: What Are the Contributions of the Selected CMH Studies?

Figure 3 shows the different contributions of the selected papers: 36.6% (106/289) of the selected studies addressed the advantages, challenges, and limitations of CMH; 24.2% (70/289) proposed tool-based solutions; and 19.7% (57/289) investigated perspectives and attitudes toward CMH. Only 3.8% (11/289) of the studies presented guidelines, and only 2.4% (7/289) provided frameworks and protocols.

### MQ5: What Are the Empirical Types of the Selected CMH Studies?

Figure 3 shows the identified empirical types of the selected papers. Overall, 52.5% (152/289) of the studies were not evaluated empirically, and 17.9% (52/289) and 12.4% (36/289) were evaluated empirically through experiments and questionnaires, respectively. Only a few publications used empirical evaluation methods such as case studies (7/289, 2.4%), focus groups (7/289, 2.4%), and interviews (6/289, 2.0%). In addition, 10.0% (29/289) of the studies implemented either a mixed method approach (17/289, 5.8%) or used a different, not largely used empirical evaluation approach (12/289, 4.1%).

### MQ6: What Are the Mental Health Issues Addressed in the CMH Literature?

**Figure 4:**
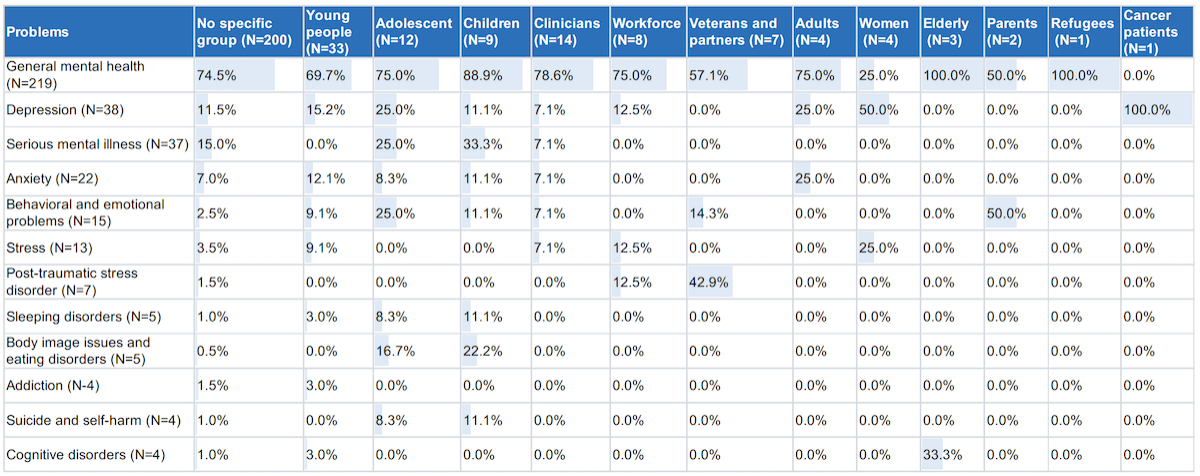
Mental health issue types versus targeted cohort. The color and the percentage represent the frequency of appearance of a problem in the selected studies dealing with a specific cohort.

Figure 4 shows the mental health issues identified in the selected studies. Most of the selected studies (219 publications) addressed mental well-being in general. A total of 38 publications addressed depression, whereas 22 publications focused on anxiety. Furthermore, 37 papers addressed serious mental illnesses such as bipolar disorder, psychosis, schizophrenia, personality disorders, dementia, panic disorders, and major depressive disorders. Among the identified mental health issues addressed in the selected papers were addiction, suicide, cognitive disorders, sleeping disorders, emotional problems, mindfulness, and productivity influenced by mental state.

### MQ7: Who Are the Targeted Cohorts in the Selected CMH Studies?

Figure 4 shows all groups identified in the studies. Most studies (200 selected publications) did not address a particular group of people. The remaining publications focused either on age-based groups, such as children, adolescents, and the elderly, or on specific groups, such as veterans, refugees, and cancer patients.

### MQ8: In Which Countries Were the Selected Empirically Evaluated Studies Conducted?

**Figure 5:**
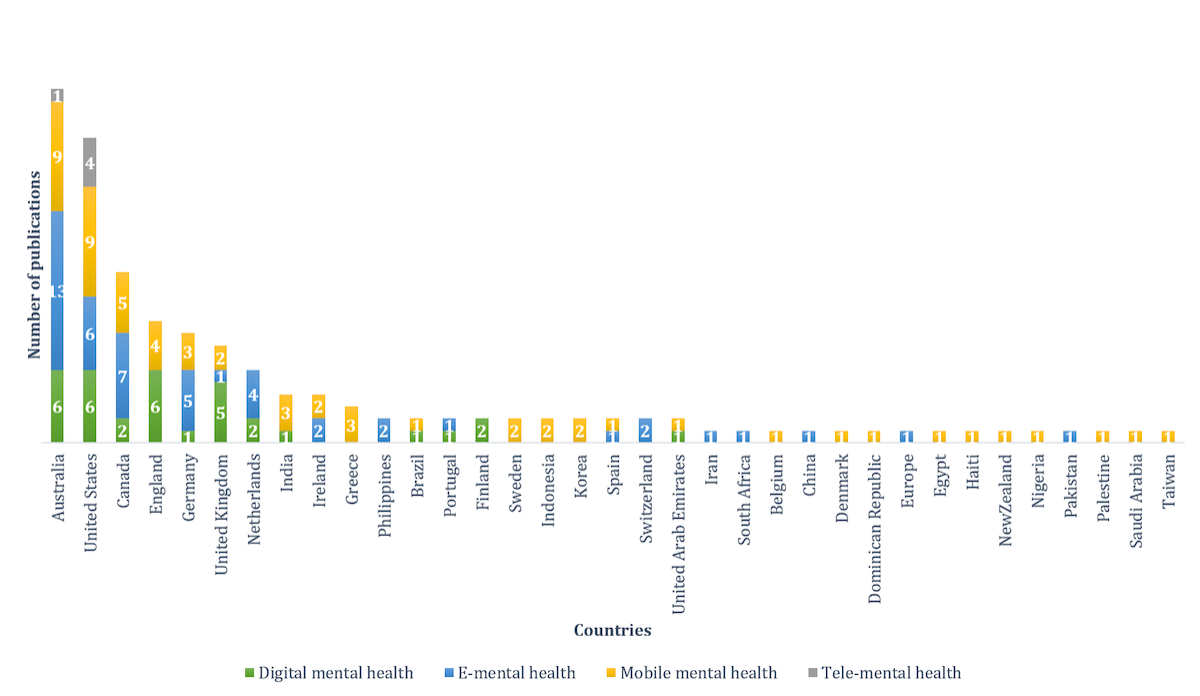
Geographical distribution of empirically evaluated studies. E-mental: electronic mental; tele-mental health: telehealth for mental health.

Figure 5 shows the geographical distribution of the selected empirically evaluated studies. Most studies were conducted in Australia, followed by the United States and Canada. Some studies included more than one country and were counted for each one.

## Discussion

### Principal Findings

#### Trends in Publication and Usage of Terms

There has been an increasing interest in CMH publications in the past decade. This can be explained by the fact that CMH research has been influenced continuously by novel technological interventions and the growth in ICT usage, especially with the appearance of Web 2.0 between 2003 and 2004 [202]. Web 2.0 introduced an architecture of participation [203], focusing on social interactions and collective intelligence [204]. These changes were accompanied by a significant increase in the number of internet users worldwide [205] as well as ownership and usage of smartphones [206] and computers [207-209]. This has also impacted the use of ICT, which has been incorporated in many fields, including transport [210], tourism [211], education [212], virtual work teams [213], and health care [214].

An increase in the number of publications in the field of CMH could have also been influenced by the changes in the general literature. There has been a shift from paper copies to electronic copies, which significantly increased the accessibility to published studies, particularly open access publications [215]. Consequently, the research evaluations performed by peers became reliant on different bibliometrics [216]. The productivity of researchers has mainly been measured by bibliometrics (eg, journal’s impact factor [217,218] and researcher’s h-index [219]), which is impacting hiring processes, academic promotion, and funding decisions [219,220]. This puts more pressure on researchers to produce more publications in the literature, particularly publications indexed in Scopus.

The earliest term used in the selected CMH studies was *telemental health*, which falls in the same group of terms as *telemedicine* and *telepsychiatry*. These terms have been used to describe remote health care and informatization of some elements of the health care process since the 1990s [221,222]. The term *telemental health* has rarely been used in recent years as *e-mental health* began emerging since 2006.

The term *eHealth* was introduced in the late 1990s. It was defined at the 7th International Congress on Telemedicine and Telecare in London at the end of November 1999 by John Mitchell as “a new term needed to describe the combined use of electronic communication and information technology in the health sector” and as “the health industry’s equivalent of e-commerce” [223]. Although previously used terms were linked to medical professionals, *eHealth* was found to be driven by nonprofessionals, namely, patients (or consumers) [224]. Many journals after the introduction of *eHealth* changed their names to include it; for example, the *Telemedicine Journal* added an “and eHealth” to its title. Nevertheless, publications were not including the term *eHealth*, and researchers preferred to use more specific terms such as *medical informatics*, *telemedicine*, and *electronic patient records*, which are considered more meaningful than the generic term *eHealth* [223]. This might explain its late and delayed emergence in the literature.

The trend of publications also showed an increasing use of the term *m-mental health*, primarily since 2010. The expansion in the use and ownership of smartphones [225] brings new opportunities for the mental health care industry to face some of the most pressing global challenges and make mental health care more accessible, faster, less expensive, and better [226]. Studies have proven the interest and willingness of people with mental health issues to try mobile solutions [73,227]. Hence, it is expected that m-mental health will continue to attract the attention of CMH researchers and practitioners.

The term *digital mental health* has been used since 2011. This term is associated with the use of recent and advanced technologies, such as sensors [228] and machine learning techniques, and it has been used as a modern synonym for *e-mental health*, especially since 2017. It must be noted that researchers use different terms to refer to the same concept, and, in many studies, authors use more than one term, which might occasionally create confusion, especially in cases in which known definitions of each term overlap. Consequently, there is a need for a broad term such as *connected mental health* to encompass the different terms used. Although *connected health* has become an established field [20], we did not find the use of such a term in mental health care. To the best of our knowledge, this is the first study that uses this term to refer to the use of ICT in mental health.

#### Publication Channels

Most of the selected papers (238/289, 82.3%) were published in scientific journals. Publishing in a rigorous scientific journal is based on a set of ethics and rules [229] that can result in the production of high-quality content. This result could have been impacted also by the choice of digital library. More journals than conference proceedings in CMH might have satisfied the criteria of Scopus indexation. There are also many scientific journals that are categorized in health informatics. This encourages researchers to conduct studies suited for journals and indicates a particular level of maturity in the field from a quantitative perspective. Articles published in journals tend to be more detailed and extensive. Conferences are also an important venue for publications. Conferences with proceedings provide fast tracking of publication to keep researchers and practitioners up to date with the latest findings. The first identified publication in the selected studies, which appeared in 2003, was a book. Books are used to introduce or provide a detailed overview of a topic.

#### Research Types and Empirical Types

The multidisciplinary nature of the CMH field and its various subareas resulted in a high ratio of exploratory research and reviews compared with other types of research, such as solution proposals, validation, and evaluation research. The diversity of the identified empirical evaluation types points to the uncertainty regarding adequate methods to evaluate CMH solutions. There is a need for evidence-based studies that will verify the efficiency of CMH interventions, create accurate results for future research, and identify challenges that need to be addressed. Most of the selected studies (152/289, 52.5%) were not evaluated empirically, which can be linked to the fact that a large number of empirical evaluation studies in the health informatics field have not been published [230]. Most of the remaining studies were empirically evaluated through experiments (52/289, 17.9%), primarily owing to their controlled environments. Case studies are more suited for sensitive problems such as mental health issues and would be more beneficial for the growth of the field, as they help identify many issues that can be faced in real-life implementation of ICT interventions. However, only 2.4% (7/289) of the selected studies were evaluated empirically through case studies. Questionnaires represented an important part of the empirical evaluation methods of the selected studies (36/289, 12.4%) used mainly in exploratory research (104/289, 35.9%) to investigate perspectives and attitudes toward the use of technology for mental health and on advantages, challenges, and limitations of CMH. Questionnaires are useful tools for data collection as they can be formulated to collect significant amounts and various types of data and can be delivered through various means to reach large groups of people.

#### Contributions of the Selected Studies

The largest number of the selected studies focused on investigating the advantages, benefits, risks, challenges, and limitations of CMH (106/289, 36.6%) as well as perspectives and attitudes of patients, clinicians, and various stakeholders toward CMH (57/289, 19.7%). An important part of the identified contributions was about *tool-based techniques* (70/289, 24.2%), whereas only a few studies represented *frameworks*, *protocols*, and *models* (14/289, 4.8%). This points to a level of ambiguity in the field as frameworks, protocols, and models are typically signs of clear understanding of a concept, its benefits, and its limitations. Tool-based techniques are usually relatively specific to one area of implementation and target a specific need, for instance, a mobile app for tracking mood changes or a website for evaluating levels of depression. Despite an increasing number of CMH publications, the types of research identified are primarily theoretical and hypothetical, indicating that researchers are still proposing their conceptual ideas for CMH. Only 4.4% (13/289) of the studies, categorized as others in Figure 3, addressed topics such as effectiveness, efficiency, usability, and quality of technological solutions for mental health care. The lack of publications on these topics is related to the aforementioned lack of empirical evaluation studies.

#### Mental Disorders and the Targeted Cohort

Most of the selected studies did not target a specific group of people (200/289, 69.2%). The remaining studies focused primarily on young people, adolescents, and children (33/89, 37%; 12/89, 13%; and 9/89, 10%, respectively). There was an overlap of age ranges among the categories, primarily owing to the existing overlapping of ages in the known definitions of the categories. The World Health Organization defines *adolescence* as the age range spanning from 10 to 19 years, *youth* as those aged between 15 and 24 years, and *young people* as the group covering the 2 categories from 10 to 24 years [231]. The adolescent group is further divided to include a subgroup of early adolescence (11-14 years) [232], which might be referred to by some authors as *children*. Targeting primarily the young category of patients can be explained by the fact that this category is characterized by the ownership of mobile devices, high usage of the internet and social networks, and familiarity with the use of technology, which makes it easy for them to become *customers* of CMH solutions.

On the basis of our findings, the *young people* category is the main target for studies on technological solutions for depression, anxiety, and related issues. These disorders are the third leading cause of death among adolescents and young adults, with a prevalence of approximately 4% in children and 10% to 20% in adolescents [233]. Other mental health issues such as body image issues, eating disorders, and sleep problems are also common among young people. Most mental health issues identified in this category could be linked to young people’s extensive use of social networks and living a digital life, making them disconnected from real life and absorbed in following other people’s lives and comparing them with their own lives. In many cases, this can cause them to feel worthless and disappointed with their lives, bodies, or achievements. Internet use has become an addiction for many young people, causing, among other problems, loss of sleep and high levels of loneliness [234] as well as many cyber-related mental problems [235].

Depression, followed by anxiety, was identified among most of the cohort groups, owing primarily to the increasing global expression of these disorders. The global population with depression increased by 18.4% between 2005 and 2015; similarly, the total number of people with anxiety increased by 14.9% between 2005 and 2015 [236]. Depression can occur as a result of other health problems, such as perinatal and postpartum depression among women [155,237] and common depression among cancer patients [238].

Many serious disorders such as schizophrenia, psychosis, and personality disorders were the subjects of many of the selected studies, pointing to the potential of using CMH not only for common mental disorders but also for more severe and complicated mental problems.

Most of the selected CMH publications addressed general mental well-being. Having more studies focusing on specific mental issues might help identify specific limitations and issues as well as highlight opportunities related to specific disorders. More studies should also focus on people in war zones as they are vulnerable to various mental problems and have more barriers to obtain mental health care, which CMH can help solve [239].

#### Countries in Which the Empirical Evaluations Were Conducted

Most empirical evaluations were conducted in developed English-speaking countries such as Australia, the United States, Canada, and England. This might be mainly owing to the advancement in research in these countries. In Australia, universities are considered *world-leading* in at least one area of research [240], and large investments are provided to research institutes [241]. There is an important research interest in health informatics in Australia [242]. Research in the United States is believed to be essential to the country’s economic growth. Therefore, the federal government has devoted a significant amount of funding for research [243]. The United States has many of the most accomplished individuals in the field of health informatics working in research projects and institutions specialized in the field [244]. Several developing countries have good ICT infrastructures and can benefit from different CMH interventions to solve several existing issues; however, limited research is conducted in developing countries. The same goes for war zones as only 2 studies addressed people in war zones: one conducted for Palestinian children [245] and the other addressed Syrian refugees in Germany, Sweden, and Egypt [106]. CMH interventions are solutions to overcome mental care barriers in developing countries and war zones. Research in this field should be shifted to countries that are most in need of CMH solutions.

#### Comparison With Related Studies

The findings of this study are aligned with the findings of other studies related to CMH:

- The identified increase in interest in the CMH literature was also reported in a literature review of e-mental health [133].
- The identified lack of empirical evaluation of the proposed solutions was reflected in the findings of a meta-analysis on digital interventions for alcohol and substance use disorders, reporting limitations in the number of randomized control trials (RCTs) in that area [117]. Another review on RCTs on the effectiveness of occupational e-mental health concluded the need for more detailed and specific effectiveness research [246]. A narrative review on digital mental health in developing countries reported a shortage of studies on the effectiveness and cost-effectiveness of the investigated internet-based programs [166]. A review on the effectiveness of mobile apps reported a lack of clinically validated evidence on the efficiency of most available apps [191].
- There was a lack of use of the term *telemental health*. Related findings were described in a review of the directions for videoconferencing and telemental health [247]. The review reported a limited number of RCTs in the field as well as methodologically flawed and limited research studies, which resulted in the research agenda of telemental health not being fully maximized [247].
- Leading countries in the empirical studies selected were Australia and the United States, which are similar to findings from other reviews. A review reported that evidence for telemental health and web-based solutions are largely led by Australia [138]. Two reviews reported that most of their selected publications were found to have mostly originated in the United States and Australia [22,133], and a study [133] found that Australian e-mental health research was higher in diversity and quantity.

### Limitations

This study has some limitations. This study did not include other digital databases. However, Scopus is widely considered to be the largest indexing digital library that includes publications from different disciplines [35]. This allowed us to identify different contributions in CMH research. It should be noted that Web of Science (WoS) was not considered as a recent study showed that slight differences exist between the scientific literature covered in Scopus and WoS Core Collection, which will result in a large number of duplicates [248].

The search strings were applied only to the titles to limit the sizes of the selection and to include only relevant and focused CMH publications, possibly omitting other candidate studies. However, we considered that if a paper’s principal focus was CMH, then at least one of the terms from the search strings could be expected to appear in the title, alleviating the risk of discarding relevant publications for this mapping study.

Other terms, particularly terms related to different mental health issues, might have been relevant for inclusion in the search strings, possibly impacting the results. However, our aim was to provide a high-level overview of existing CMH literature.

Other classification criteria might have been relevant to extracting further information from the selected studies, possibly affecting our findings. However, the principal aim of this systematic mapping study was to provide an overview of CMH literature, and the process and criteria presented in the *Methods* section fit this purpose.

The final study selection depended on the first two authors, ND and SO, who conducted the search process. The first author conducted a preliminary search in Scopus and constructed a preliminary selection of publications. The second author revised the selection. If a disagreement arose between the two authors on the exclusion or inclusion of a paper, then a discussion took place until an agreement was reached. It should be noted that the two authors have experience in conducting mapping studies and systematic literature reviews [228,249,250].

### Conclusions

This paper conducted a systematic mapping study of 289 publications indexed in Scopus and provided an overview of the available literature on CMH research. CMH has the potential to overcome some of the mental health care delivery barriers by introducing and exploiting ICT in the process of mental health care. A total of 289 publications were selected, analyzed, and classified. The results showed that CMH is a promising research field that has gained increasing attention from researchers over the years. The frequency of the selected publications was influenced by the continuous developments in digital media and the use of ICT as well as the changes in literature evaluation methods, which mainly relied on bibliometrics. The results also showed that most of the selected CMH literature addressed general mental well-being; depression and anxiety were the mental disorders most addressed in studies on specific mental issues, which is in accordance with their global prevalence; young people were the most targeted cohort group as they are more familiar with digital solutions; exploratory research and reviews were the dominant research types found in the selected literature, which indicates that researchers are more focused on exploring possible venues of implementation of ICT in mental health care and constructing an understanding of the field; and most of the selected studies were not empirically evaluated. Moreover, the results showed that selected studies that were empirically evaluated were mostly conducted in developed countries. Screening of the selected studies that were reviews showed that they targeted specific cohort groups, specific mental disorders, specific types of solutions, or specific terms. To the best of our knowledge, this is the first mapping study that addresses the field of CMH as a whole, including all relevant terms and without excluding any specific criteria. On the basis of our findings, we recommend the following to researchers:

- Shift attention to provide evidence-based solutions and studies, and empirically evaluate existing solutions in future CMH studies.
- Focus on specific mental issues and cohort groups to better identify possible issues and limitations of the field.
- Investigate CMH solution implementation in developing countries and war zones, which experience various mental care delivery barriers.
- Use *connected mental health* as an englobing term for the exploitation of the different types of ICT in mental health care.

Practitioners can use this study to find tool-based studies for specific cohorts and/or mental disorders. They can also find studies related to the attitudes and behaviors of CMH users.

We believe that our study will provide researchers and practitioners with relevant information regarding the existing CMH literature as well as recommendations for future publications. For future work, we intend to develop a conceptual framework for sustainable CMH solutions.

## Appendix

Multimedia Appendix 1 Classification results.

## Abbreviations

CMH: connected mental health
COVID-19: coronavirus disease
EC: exclusion criteria
e-mental: electronic mental
ICT: information and communication technologies
m-mental: mobile mental
MQ: mapping question
RCTs: randomized control trials
WoS: Web of Science
